# Blockchain Personal Health Records: Systematic Review

**DOI:** 10.2196/25094

**Published:** 2021-04-13

**Authors:** Hao Sen Andrew Fang, Teng Hwee Tan, Yan Fang Cheryl Tan, Chun Jin Marcus Tan

**Affiliations:** 1 SingHealth Polyclinics Singapore Singapore; 2 National University Health System Singapore Singapore; 3 SingHealth Community Hospitals Singapore Singapore

**Keywords:** blockchain, personal health records, electronic health records, distributed ledger, systematic review

## Abstract

**Background:**

Blockchain technology has the potential to enable more secure, transparent, and equitable data management. In the health care domain, it has been applied most frequently to electronic health records. In addition to securely managing data, blockchain has significant advantages in distributing data access, control, and ownership to end users. Due to this attribute, among others, the use of blockchain to power personal health records (PHRs) is especially appealing.

**Objective:**

This review aims to examine the current landscape, design choices, limitations, and future directions of blockchain-based PHRs.

**Methods:**

Adopting the PRISMA (Preferred Reporting Items for Systematic Reviews and Meta-analyses) guidelines, a cross-disciplinary systematic review was performed in July 2020 on all eligible articles, including gray literature, from the following 8 databases: ACM, IEEE Xplore, MEDLINE, ScienceDirect, Scopus, SpringerLink, Web of Science, and Google Scholar. Three reviewers independently performed a full-text review and data abstraction using a standardized data collection form.

**Results:**

A total of 58 articles met the inclusion criteria. In the review, we found that the blockchain PHR space has matured over the past 5 years, from purely conceptual ideas initially to an increasing trend of publications describing prototypes and even implementations. Although the eventual application of blockchain in PHRs is intended for the health care industry, the majority of the articles were found in engineering or computer science publications. Among the blockchain PHRs described, permissioned blockchains and off-chain storage were the most common design choices. Although 18 articles described a tethered blockchain PHR, all of them were at the conceptual stage.

**Conclusions:**

This review revealed that although research interest in blockchain PHRs is increasing and that the space is maturing, this technology is still largely in the conceptual stage. Being the first systematic review on blockchain PHRs, this review should serve as a basis for future reviews to track the development of the space.

## Introduction

### Background

Personal health records (PHRs) are a form of electronic health records (EHRs). PHRs are unique in that patients themselves can access, manage, and share their health information [1]. The benefits of PHRs include patient empowerment, which leads to improved outcomes and reduced health care costs [2,3]. Although interest in PHRs has been increasing, their adoption remains low [4,5]. One of the oft-cited reasons is related to privacy and security concerns owing to an increasing trend of health information breaches [6,7]. Another reason is the lack of perceived usefulness to patients [7].

Blockchain technology was introduced through Bitcoin in 2008 [8]. It is considered a general-purpose technology and has since been successfully applied across several different industries [9,10]. In the health care industry, EHRs were found to be the most commonly used case for blockchain applications [11-14]. Compared with conventional data management methods that rely on on-premise data servers or third-party cloud services, blockchain’s distributed ledger technology offers a novel alternative. This could potentially address the privacy and security concerns surrounding EHRs [15]. Specifically for application to PHRs, blockchain also has the ability to decentralize control and incorporate incentive mechanisms through smart contracts, which can further entice its general use and increase adoption [16]. These advantages, among others, have motivated efforts to test the feasibility and implement blockchain PHRs [17-19].

The research space in which EHRs and blockchain intersect is still in its infancy, with the first blockchain EHR introduced in 2016 [20]. Systematic reviews covering this space so far have considered EHRs as a collective entity. Mayer et al [21] provided an overview of the ecosystem of blockchain EHRs while also proposing a taxonomy for the space. Shuaib et al [22] looked at the main areas of focus when implementing a blockchain EHR and the remaining issues to be addressed, whereas Vazirani et al [23] assessed the feasibility of blockchain as a method of managing health care records efficiently.

Given that one of the inherent properties of blockchain is its decentralized nature, in which data ownership is placed in the hands of individual users, some have proposed that blockchain may be more suitably applied to PHRs specifically rather than EHRs in general [19,24-26]. In this paper, we aim to systematically review the following: (1) the current landscape and trends of blockchain-based PHRs (blockchain PHRs), (2) the attributes of various blockchain PHRs that have been described, and (3) the current limitations and future directions for blockchain PHRs. To the best of our knowledge, this is the first systematic review examining blockchain with PHRs. We hope that this review will serve as a useful reference, especially for those intending to develop a blockchain PHR and for future reviews in this area.

To provide more context for subsequent sections of this paper, we will first explain pertinent blockchain concepts and take the opportunity to introduce some terminology specific to blockchain. This is by no means an exhaustive explanation of blockchain.

### What Is a Blockchain?

**Figure 1:**
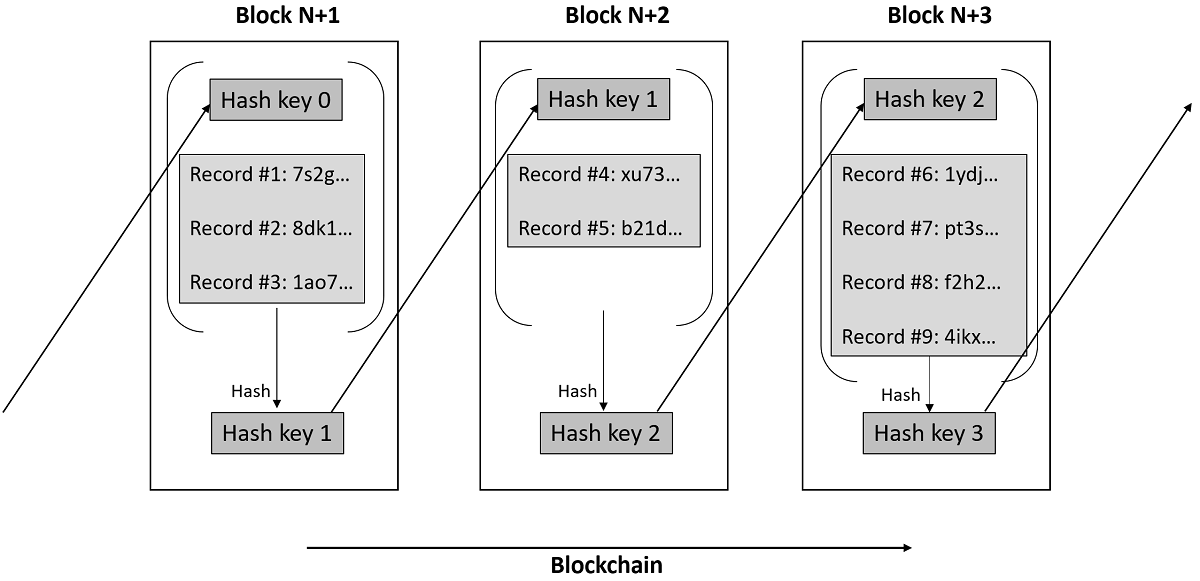
Illustration of how blocks of data are linked together in a blockchain through hashing. To add a new record (eg, Record #7) to the blockchain, this is first grouped with other records (Records #6, #8, and #9). The group of records is then combined with a hash key from the previous block (hash key 2) and then put through a hashing algorithm to produce a new hash key (hash key 3). The new records, along with hash keys 2 and 3, are now part of a new block (Block N+3) that has been added to the blockchain. This process continues as new records are added.

A blockchain can be thought of as a shared (or distributed) database that is spread across multiple sites and participants. For new data to be added to a blockchain, they are first compiled into a *block*, which is simply a collection of records to be added to the database. The block is then combined with some data (a *hash key*) from the previous block through a cryptographic technique called *hashing* before it is added. As it combines the previous block’s hash key, each new block is tied to all its predecessors in the form of a chain—hence the term *blockchain* (Figure 1).

### Types of Blockchains and Their Properties

Before data can be added to a blockchain, its users need to agree or reach *consensus*. This is achieved through a *consensus algorithm*. A well-known consensus algorithm is the proof of work (PoW) algorithm. PoW is used in the Bitcoin and Ethereum blockchain network protocols [8,27]. In the PoW algorithm, users (also known as *miners*) compete in computational tasks to reach consensus. The winning miner of each block’s task is usually given a reward [28].

Blockchains can be classified into the following three types, depending on which participants are allowed in the consensus algorithm [28]:

1. Public: anyone can participate in the consensus algorithm. Examples include Bitcoin and Ethereum [8,27].
2. Consortium: a select (or permissioned) group of entities can participate in the consensus algorithm. Examples include Hyperledger Fabric (HF), Quorum, and Corda [29-31].
3. Private: only a single entity operates the consensus algorithm and controls the addition of new data.

Public blockchains are sometimes referred to as permissionless blockchains, whereas consortium and private blockchains are collectively termed permissioned blockchains.

The three types of blockchains differ in the following properties:

1. Decentralization: unlike traditional databases that are owned by a specific entity, a decentralized blockchain can allow every user to own the data collectively. Using the illustration in Figure 1 as an example, a decentralized blockchain would contain all the records, but only one user owns records #1, #3, #4, #6, and #8, and another user separately owns records #2, #5, #7, and #9.
2. Immutability: because of the underlying chain structure, once data have been added to the blockchain, they cannot be tampered with. Changing a record would alter the hash key and effectively cause a break in the chain.
3. Transparency (with privacy): the entire blockchain can be made publicly viewable while preserving privacy by masking each individual record using cryptography. To unmask one’s own records, a private key is required.

Table 1 provides a summary of the different types of blockchains and their properties, with an example of each type in the health care setting.

**Table 1:** Comparison of public, consortium, and private blockchains.

Variables	Type of blockchain		
	Public	Consortium	Private
Participation in consensus protocol	Anyone	Select (or permissioned) group of entities	A single entity
Decentralization	Yes	Partial	No
Immutability	Tamperproof	Could be tampered with	Could be tampered with
Transparency	Public	Can be public or restricted	Can be public or restricted
Example in health care setting	A transnational, open EMR^a^ system in which anyone (eg, health care institutions, patients) may choose to contribute their own resources to maintain health records of all patients who use the EMR system	A national EMR system in which selected health care institutions collectively maintain the health care records of their patients	An institution-based EMR system in which only a single institution maintains the health records of its own patients

^a^EMR: electronic medical record.

### Scalability and Smart Contracts

Finally, we will briefly explain the two concepts of *scalability* and *smart contracts*, which will be relevant to subsequent parts of this paper.

Scalability refers to the capacity of a blockchain to store and process transactions. It generally relates to the size and frequency of transactions a blockchain can handle. For example, Bitcoin’s block size is limited to 1 megabyte, and each block is added every 10 minutes. This translates to a rate of approximately 7 transactions per second. Various solutions have been proposed to improve scalability. One such solution is to store data *off-chain* (instead of on-chain), and another solution is to use *side-chains* (linked to the main chain) to enable larger transaction volumes to be processed in parallel. Given that health care data are estimated to reach as much as 2314 exabytes generated yearly by 2020, it is crucial for almost all blockchain-based health care applications to achieve a certain level of scalability [32].

Smart contracts are programmable computer rules. Blockchain is a digital database that allows for the implementation of smart contracts, which can be automatically triggered to execute when predefined conditions are satisfied. For example, a smart contract can be programmed to issue tokens on the blockchain each time a user records his or her blood pressure. These tokens can then be used to pay for health care services. Such smart contracts can thus potentially be used to enable incentive structures to encourage certain positive user behaviors.

In this systematic review, particularly focused on the blockchain component of blockchain PHRs, we will pay particular attention to the (1) type of blockchain, (2) scalability solutions, and (3) smart contract–based incentive structures.

## Methods

### Study Design

While conducting and reporting this systematic literature review, the guidelines described in the PRISMA (Preferred Reporting Items for Systematic Reviews and Meta-analyses) statement were adopted [33]. This type of literature review was selected because the goal was to identify articles on blockchain PHRs and to summarize the current landscape, design choices, limitations, and future directions. Unlike a meta-analysis, this review did not require any data synthesis. Quality assessment was not performed because the intention was to achieve a collective understanding of the efforts and ideas rather than judging the quality of various blockchain PHRs.

The presented systematic review was carried out by defining the following activities:

1. Research questions
2. Search strategy
3. Article selection
4. Data abstraction

#### Research Questions

For this review, there were 3 research questions we aimed to address:

1. What are the current landscape and trends of blockchain PHRs in terms of interest groups, geography, and maturity level?
2. What were the key design decisions made for the blockchain PHRs described?
3. What are the current limitations faced by blockchain PHRs and future directions?

#### Search Strategy

The following search string was used: “blockchain” AND (“health record*” OR “medical record*” OR “*EHR*” OR “*EMR*” OR “*PHR*”). Articles in the following databases were searched: (1) ACM, (2) IEEE Xplore, (3) MEDLINE, (4) ScienceDirect, (5) Scopus, (6) SpringerLink, (7) Web of Science, and (8) Google Scholar. For databases whose search engines did not enable the use of wildcards, the search was widened to include abstracts and keywords, and Microsoft Excel was subsequently used to filter the returned list by applying the search string to the titles.

As the space is still in its infancy stage, Google Scholar was included as a search database to incorporate relevant gray literature in this review. This decision was supported by systematic reviews by Holbl et al [34] and Kuo et al [35] on blockchain in the health care domain, which had found valuable information residing in gray literature.

#### Article Selection

Once the articles were obtained, we applied the following inclusion and exclusion criteria to select articles for the final review. The inclusion criteria were as follows: a health record system that had (1) a patient-facing component and (2) used blockchain in its health record system. The exclusion criteria were as follows: (1) duplicate articles, (2) review articles, (3) articles that did not have full text available, and (4) articles whose full text was not in English.

The selection was performed in a stepwise manner. First, duplicate articles returned from multiple databases were excluded. Second, the titles of the articles were reviewed and those that were not relevant to the topic were discarded. Third, the abstracts of the articles were reviewed and those whose main focus was not on blockchains and EHR or PHR and those that were review articles were also discarded. Those that looked at EHRs at this stage were retained because some EHRs would have a patient-facing component but might not have been explicitly mentioned in the title or abstract. Finally, the full text was reviewed and those that did not have a PHR element in the EHR were discarded. At this stage, those that did not have full text available or whose full text was not in English were also excluded.

#### Data Abstraction

For data abstraction, a standardized data collection form was developed using Microsoft Excel. A full-text review of each selected study was performed independently by 3 reviewers who are knowledgeable about blockchain and health records. For discrepancies in the abstracted data, the reviewers performed a repeat review of the articles together to reach a consensus.

For the interest groups, author affiliations, publishers, and publications were used as a proxy. As this space is situated at the intersection of computer science (CS), engineering, and medicine, we classified the publications into either (1) CS or engineering, (2) medical, or (3) general. For maturity level, the classification used by Chukwu et al [12] was modified, and the projects were classified as *concept/model/framework*, *prototypes*, and *pilots or implementations*. A prototype was considered to have both a working front-end and back-end system, and a pilot or implementation had to be a product that was released for use in the real world. If an article described systems at multiple levels of maturity (eg, a framework and a prototype), only the more mature level described was abstracted.

Many design choices must be made when developing a blockchain PHR. To keep this review manageable, the review focused on high-level design decisions [36]. To ensure a comprehensive list of possible design parameters, the *PHR taxonomy* proposed by Roehrs et al [37] and *EHR in a Blockchain taxonomy* proposed by Mayer et al [21] were used as starting points. Next, through a consensus-driven process of elimination, 10 design parameters were selected for abstraction. These were (1) blockchain type, (2) data storage, (3) scaling solution, (4) incentive smart contract, (5) PHR type, (6) data owner, (7) read and write ability, (8) semantic standards, (9) privacy standards, and (10) user interface (UI).

For limitations and future directions, the issues and areas for improvement brought up across the articles reviewed were identified, consolidated, and presented as a list of unique issues. We did not delve into a more in-depth analysis such as ranking the unique issues because the frequency of mention was not necessarily associated with importance or criticality. Moreover, the articles may not have fully listed all their limitations, as it was not their primary aim.

In total, 23 data elements were extracted from each article. Table 2 provides a complete list of the extracted data elements and a description of each element.

**Table 2:** List of data elements extracted from the selected articles.

Types of data elements			Description
**General**			
	Author	First author’s last name	
	Title	Title of the article	
	Year	Publication year of the article	
	Country	First author’s affiliated country	
	Type	Type of article (eg, journal article, conference paper, book chapter, and whitepaper)	
	Publisher	Name of publisher of the article	
**Blockchain**			
	Name of blockchain PHR^a^	Name of the blockchain PHR (if any)	
	Maturity	Maturity level of the blockchain PHR described (ie, concept/framework, prototype, and pilot/implementation)	
	Blockchain type	Type of blockchain (ie, public, consortium, private, or a combination)	
	Blockchain name	Name of the blockchain used (if any)	
	Data storage	Type of data storage mechanism (ie, on-chain, off-chain, or hybrid)	
	Scaling solution	Type of scaling solution used in the blockchain PHR (if any)	
	Incentive smart contract	Was a smart contract used to incentivize use of the blockchain PHR? (yes or no)	
**PHR**			
	PHR type	Type of PHR (ie, standalone or tethered to an existing EMR^b^ system)	
	Data owner	Party that owned the data from the blockchain PHR (ie, patient, provider, or both)	
	Read-write access (for patients)	Was the patient given read and/or write access in the blockchain PHR? (yes or no)	
	Read-write access (for providers)	Was the provider given read and/or write access in the blockchain PHR? (yes or no)	
	Read-write access (for other parties)	Were other parties given read and/or write access in the blockchain PHR? (yes or no)	
	Semantic standard	Type of semantic standard adopted (eg, HL-7^c^ and FHIR^d^)	
	Privacy standard	Type of privacy standard adopted (eg, HIPAA^e^ and GDPR^f^)	
	User interface	Modality of accessing the blockchain PHR (ie, web, mobile, or desktop application)	
**Additional**			
	Limitations	Current limitations of the blockchain PHR	
	Future directions	Future directions and opportunities described	

^a^PHR: personal health record.

^b^EMR: electronic medical record.

^c^HL-7: health level 7.

^d^FHIR: Fast Healthcare Interoperability Resource.

^e^HIPAA: Health Insurance Portability and Accountability Act.

^f^GDPR: General Data Protection Regulation.

## Results

### Overview of Articles

**Figure 2:**
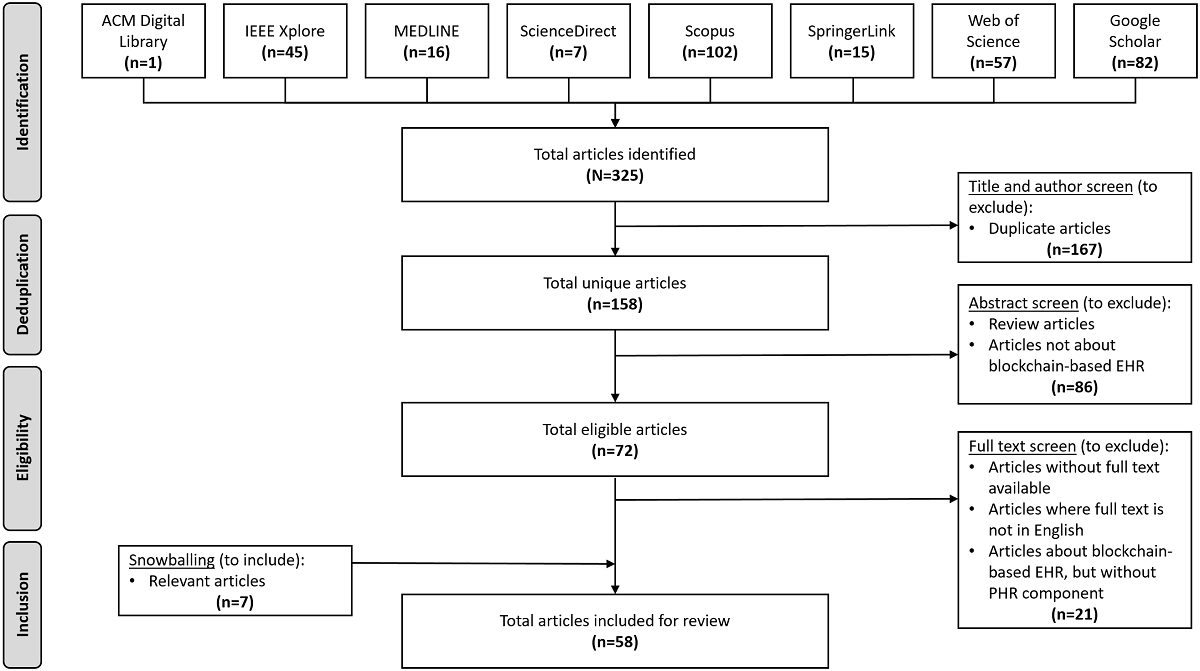
PRISMA (Preferred Reporting Items for Systematic Reviews and Meta-analyses) flow diagram of the article selection process. The title and author screen involved removing duplicate articles that had the same title and authors. The abstract screen involved reviewing article abstracts to remove review articles and those not related to blockchain and electronic health records. The full text screen involved reviewing the full articles to exclude those that did not meet the inclusion and exclusion criteria, and those whose full text was not available or in English. ACM: Association of Computing Machinery; EHR: electronic health record; IEEE: Institute of Electrical and Electronics Engineers; PHR: personal health record.

The search performed on July 6, 2020, yielded 325 articles, of which 158 were unique articles. From the article selection process, 51 articles were selected for review. An additional 7 articles were added via snowballing (review of the references from the included articles) of the full texts screened (Figure 2).

A total of 58 studies were included in the final review [17,19,37-92]. The complete list of articles, with identifiers used in this study, are presented in Table 3. The completed data collection form for these articles can be found in Multimedia Appendix 1. An overview of the articles with the publication year, publisher, article type, country, and interest group is presented in Multimedia Appendix 2 [17,19,37-92].

**Table 3:** List of articles included in the final review.

Article identifier	Authors	Article title
A01	Burniske [37]	How blockchain technology can enhance EHR^a^ interoperability
A02	McFarlane et al [38]	Patientory: A Healthcare Peer-to-Peer EMR^b^ Storage Network
A03	Roehrs et al [39]	OmniPHR: A distributed architecture model to integrate personal health records
A04	Badr et al [40]	Multi-tier blockchain framework for IoT^c^-EHRs systems
A05	Boiani [41]	Blockchain based electronic health record management for mass crisis scenarios: A feasibility study
A06	Chen et al [42]	Blockchain-Based Medical Records Secure Storage and Medical Service Framework
A07	Dagher et al [43]	Ancile: Privacy-preserving framework for access control and interoperability of electronic health records using blockchain technology
A08	Dubovitskaya et al [44]	Secure and Trustable Electronic Medical Records Sharing using Blockchain
A09	Gebremedhin [45]	Blockchain as a Technology to Facilitate Privacy and Better Health Record Management
A10	Lippman et al [46]	MedRec: Patient Control of Medical Record Distribution
A11	Medicalchain [47]	Medicalchain
A12	Rouhani et al [48]	MEDICHAIN: A Secure Decentralized Medical Data Asset Management System
A13	Thwin and Vasupongayya [49]	Blockchain Based Secret-Data Sharing Model for Personal Health Record System
A14	Vora et al [50]	BHEEM^d^: A Blockchain-Based Framework for Securing Electronic Health Records
A15	Zhang and Poslad [51]	Blockchain Support for Flexible Queries with Granular Access Control to Electronic Medical Records (EMR)
A16	Abouzahra [52]	Using blockchain technology to enhance the use of personal health records
A17	Alkhushayni et al, [53]	Blockchain technology applied to electronic health records
A18	Ray Chawdhuri [54]	Patient Privacy and Ownership of Electronic Health Records on a Blockchain
A19	Ciampi [55]	A Blockchain Architecture for the Italian EHR System
A20	Daraghmi et al [56]	MedChain: A Design of Blockchain-Based System for Medical Records Access and Permissions Management
A21	Donawa et al [57]	Scaling Blockchains to Support Electronic Health Records for Hospital Systems
A22	Hang et al [58]	A novel EMR integrity management based on a medical blockchain platform in hospital
A23	Harika et al [59]	Blockchain technology for managing an architectural model of decentralized medical record
A24	Huang et al [60]	MedBloc: A Blockchain-Based Secure EHR System for Sharing and Accessing Medical Data
A25	Hylock and Zeng [61]	A Blockchain Framework for Patient-Centered Health Records and Exchange (HealthChain): Evaluation and Proof-of-Concept Study
A26	Jiang et al [62]	Patients-Controlled Secure and Privacy-Preserving EHRs Sharing Scheme Based on Consortium Blockchain
A27	Koushik et al [63]	Performance Analysis of BlockChain-based Medical Records Management System
A28	Lee [64]	PHR^e^ system using blockchain technology
A29	MediBloc [65]	MediBloc Technical Whitepaper
A30	MediLOT [66]	MediLOT Whitepaper
A31	Nchinda et al [67]	MedRec: A Network for Personal Information Distribution
A32	Nguyen et al [68]	Blockchain for Secure EHRs Sharing of Mobile Cloud Based E-Health Systems
A33	Park et al [17]	Is Blockchain Technology Suitable for Managing Personal Health Records? Mixed-Methods Study to Test Feasibility
A34	Rajput et al [69]	EACMS: Emergency Access Control Management System for Personal Health Record Based on Blockchain
A35	Reen et al [70]	Decentralized patient centric e-Health record management system using blockchain and IPFS^e^
A36	Sangeetha [71]	Electronic Health Record System using Blockchain
A37	Shahnaz et al [72]	Using Blockchain for Electronic Health Records
A38	Shekhawat [73]	Cloud-chain: Revamp Health Record System Using Blockchain
A39	Thwin and Vasupongayya [74]	Blockchain-Based Access Control Model to Preserve Privacy for Personal Health Record Systems
A40	Tian [75]	Blockchain-based secure medical record sharing system
A41	Toshniwal et al [76]	PACEX: Patient-centric EMR exchange in Healthcare Systems using Blockchain
A42	Wang et al [77]	Blockchain-Based Personal Health Records Sharing Scheme With Data Integrity Verifiable
A43	Wang et al [78]	Cloud-Assisted EHR Sharing with Security and Privacy Preservation via Consortium Blockchain
A44	Wu and Du [79]	Electronic medical record security sharing model based on blockchain
A45	Al Goni et al [80]	A P2P Optimistic Fair-Exchange Scheme for Personal Health Records Using Blockchain Technology
A46	Arunkumar and Kousalya [81]	Blockchain-Based Decentralized and Secure Lightweight E-Health System for Electronic Health Records
A47	Aswin et al [82]	Design of AYUSH: A blockchain-based health record management system
A48	Cao et al [83]	Hybrid blockchain–based privacy-preserving electronic medical records sharing scheme across medical information control system
A49	Charanya et al [84]	Sefra: A secure framework to manage eHealth records using blockchain technology
A50	Kavathekar and Patil [85]	Data sharing and privacy-preserving of medical records using blockchain
A51	Kim et al [86]	Design of Secure Protocol for Cloud-Assisted Electronic Health Record System Using Blockchain
A52	Kung et al [87]	Personal Health Record in FHIR^f^ Format Based on Blockchain Architecture
A53	Lee et al [19]	An Architecture and Management Platform for Blockchain-Based Personal Health Record Exchange: Development and Usability Study
A54	Sharma et al [88]	Secure Cloud Storage Architecture for Digital Medical Record in Cloud Environment using Blockchain
A55	Sharma and Balamurugan [89]	Preserving the Privacy of Electronic Health Records using Blockchain
A56	Tith et al [90]	Application of Blockchain to Maintaining Patient Records in Electronic Health Record for Enhanced Privacy, Scalability, and Availability
A57	Verdonck and Poels [91]	Architecture and value analysis of a blockchain-based electronic health record permission management system
A58	Wu et al [92]	Secure Personal Health Records Sharing Based on Blockchain and IPFS^g^

^a^EHR: electronic health record.

^b^EMR: electronic medical record.

^c^IoT: internet of things.

^d^BHEEM: Blockchain-based framework for efficient storage and maintenance of electronic health records

^e^PHR: personal health record.

^f^FHIR: Fast Healthcare Interoperability Resource.

^g^IPFS: Interplanetary File System.

### Current Landscape and Trends of Blockchain PHRs

#### Interest Group

**Figure 3:**
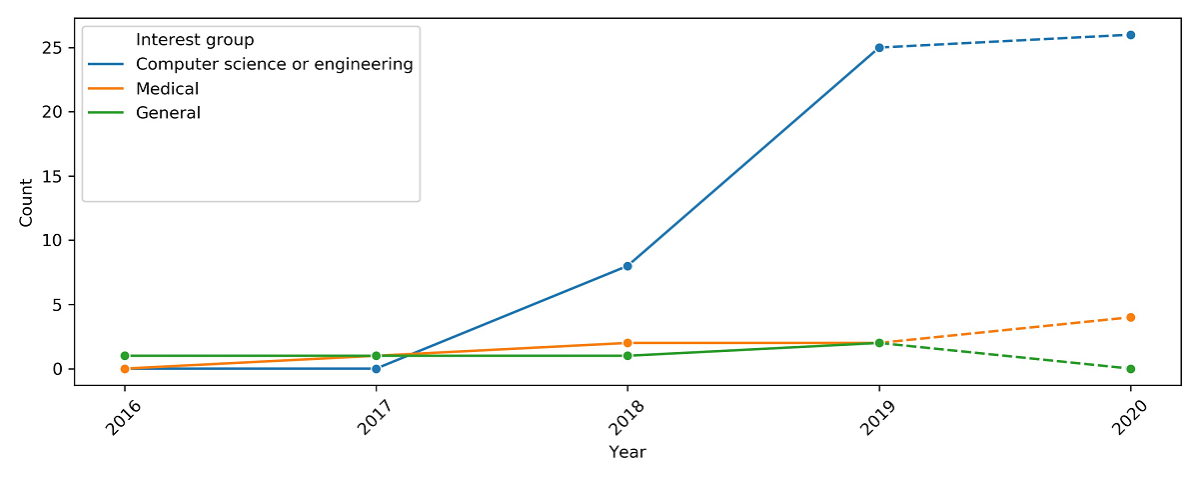
Trend of blockchain personal health record articles by interest group. The trend from 2019 to 2020 (represented by dashed lines) is a projection because only data from the first half of the year 2020 was available at the time of the search. Count refers to the number of articles published in that year.

The level of academic interest in the space has been rising, supported by an increasing trend in the number of published articles since 2016. In terms of interest groups, 45 articles were CS-or engineering-related publications or from CS- or engineering-related authors. Seven were published in medical journals, all of which were related to medical informatics. Of the 6 remaining articles that were classified as *General*, 5 were whitepapers. The articles from the CS or engineering interest group showed a sharp rise from 2017 to 2019 and may have started to plateau, whereas those from medical journals have been following a gradual, steady increase since 2016 (Figure 3).

#### Geographic Distribution

**Figure 4:**
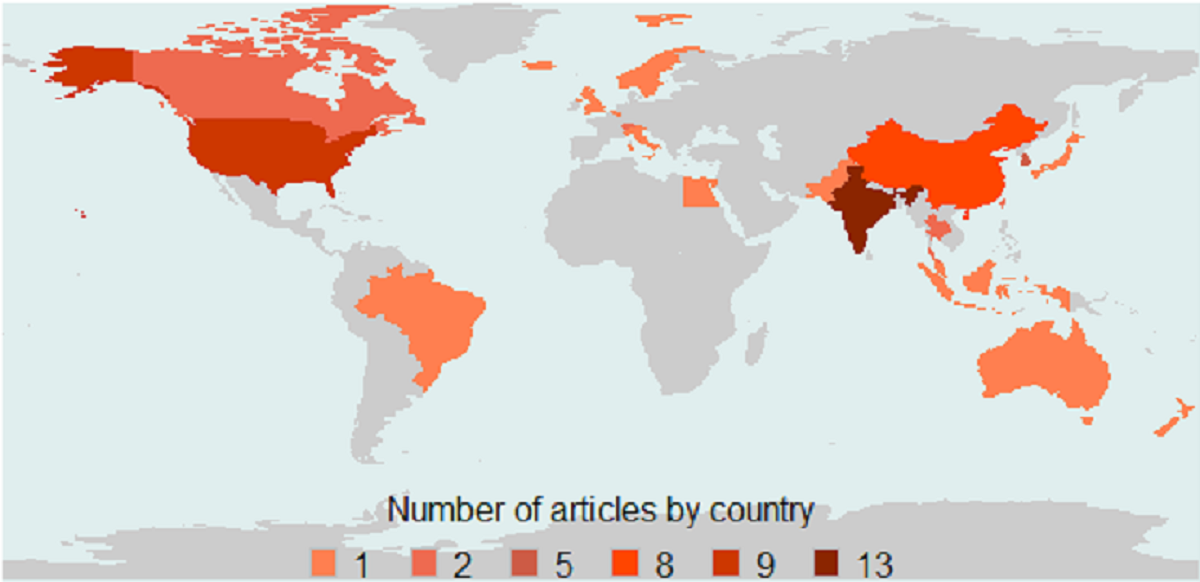
Distribution of articles published by geography. The number of articles refers to the total number of articles selected for the final review.

**Figure 5:**
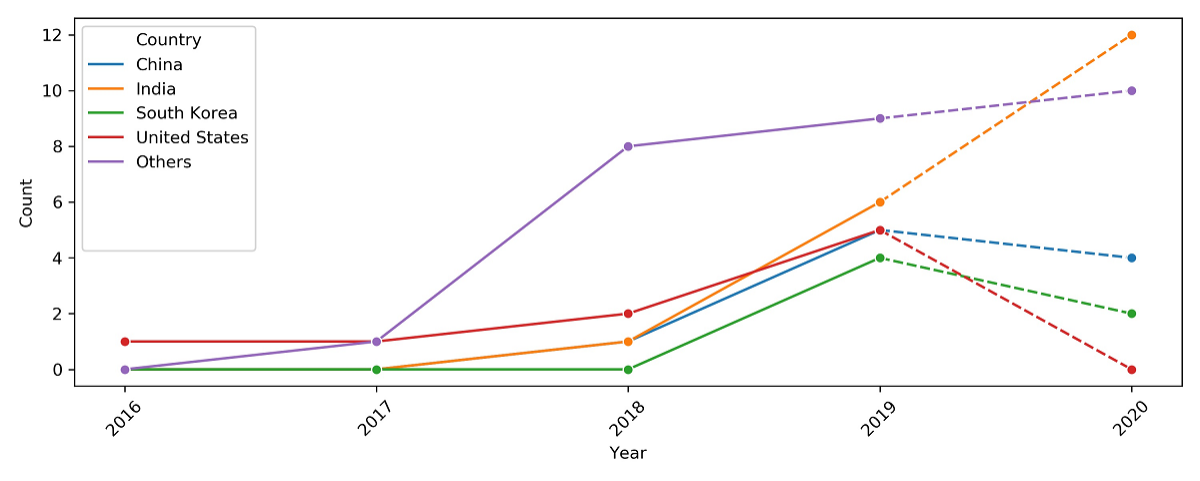
Trend of blockchain personal health record articles by country. Only countries with 5 or more articles in the final review were plotted individually. The other countries were grouped under an Others category. The trend from 2019 to 2020 (represented by dashed lines) is a projection because only data from the first half of the year 2020 was available at the time of the search. Count refers to the number of articles published in that year.

The articles originated from 23 different countries. The majority were from India (n=13), United States (n=9), China (n=8), and South Korea (n=5), with Canada, Switzerland, Taiwan, and Thailand having 2 articles each and the remaining countries having 1 article each (Figure 4). Although the research interest in blockchain PHR is multinational, there clearly are a few countries that are leading the pack. Among these leading countries, there has been an increasing number of publications from India over the years, whereas China, South Korea, and the United States have shown a slowing trend. Apart from these countries, the aggregated output from the rest of the countries is also increasing (Figure 5).

#### Maturity Level

**Figure 6:**
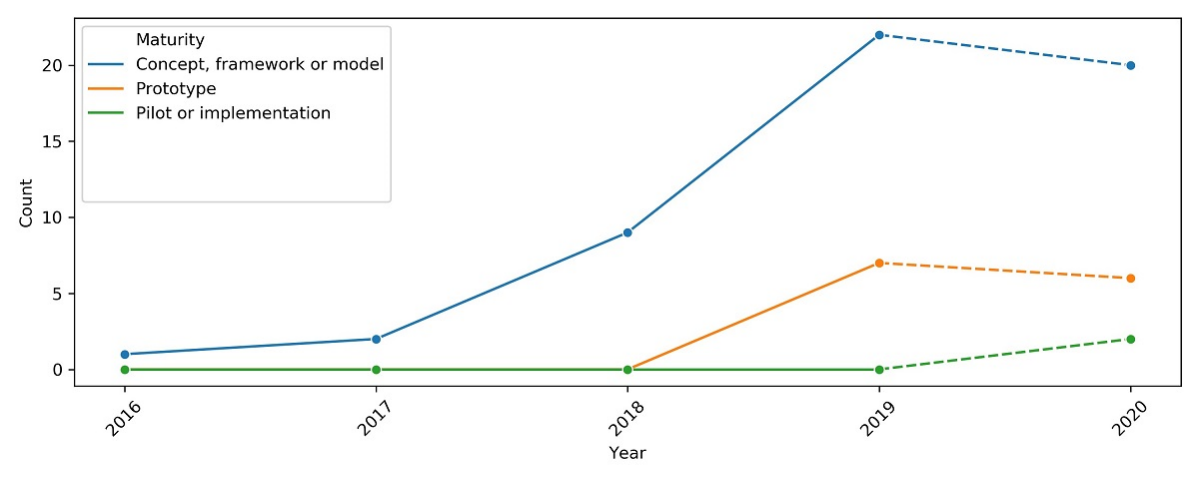
Trend of blockchain personal health record maturity. Note that the trend from 2019 to 2020 (represented by dashed lines) is a projection because only data from the first half of the year 2020 was available at the time of search. Count refers to the number of articles published in that year (2018).

The blockchain PHR space is maturing, with the proportion of articles describing prototypes showing an upward trend (Figure 6). In addition, the first paper to describe an implementation was also published in the first half of 2020 by Lee et al [19]. Their blockchain PHR implementation was deployed across Southeast Asia via an information network and became the first PHR management platform for cross-regional medical data exchange.

### Key Design Choices for Blockchain PHRs

#### Blockchain Attributes

Most blockchain PHRs are described using a private (n=24) or consortium (n=22) blockchain, whereas 4 others used a public-permissioned hybrid design. Only 4 cases of using a public blockchain were described. In the remaining 4 cases, the blockchain type was not clearly stated. An Ethereum-based blockchain was the most commonly used (n=26), with HF being the next most common (n=20). Among these, 3 articles used both Ethereum and HF.

For data storage, the majority used off-chain data storage (n=40), 14 stored EHR data on-chain, and 4 described hybrid data storage. For off-chain storage, 10 articles, all from 2019 onward, used the Interplanetary File System (IPFS). In terms of other scaling solutions, 9 articles considered new consensus algorithms such as Proof-of-Authority, 4 used a tiered-chain architecture, and 1 used both side-chain and algorithmic methods to improve the blockchain scaling capacity.

Among the articles, 5 described an incentive structure in the blockchain PHR using smart contracts. Four of these were whitepapers, which proposed incentivizing stakeholders through the issuing of tokens (digital currency of value) from smart contracts. In these cases, once an action warranting compensation had taken place, the smart contract automatically triggered the issuance of tokens. Table 4 provides additional details of the tokens and how they can be earned and used as part of the incentive structure. Unlike the others, Daraghmi et al [56] proposed a novel, nonmonetary incentive. Their system kept score using *degrees* based on the effort in maintaining the quality of records and creating new blocks. Those with higher degrees would have a lower probability of performing the computation task of creating new blocks. In this way, it is meant to achieve fairness and sustainability of the system.

**Table 4:** Incentive structure proposed by blockchain personal health record systems.

Article identifier	Token name	Party compensated	Compensation	Token use
A02	Patientory issued tokens	Provider	On the basis of how effective provider ensures improvement in care quality and outcomes	Renting storage space on the platform Execution of smart contracts
A11	MedToken	Patients	Sharing of personal health data on health care data marketplace	Lower insurance premiums Payment for use of applications (eg, tele-consultations)
A29	MediBloc coin	Provider	Performing computational task of producing blocks	Tradable for monetary value
A30	LOT^a^ token	Patients	Contribute data Compliance to health care recommendations	Analytics services (eg, personalized health reports) Payment to retail and pharmaceutical partners

^a^LOT: token used in the MediLOT system.

#### PHR Attributes

A total of 18 articles described a tethered blockchain PHR that interfaced with an existing electronic medical record (EMR) system. All of these were of the *Concept/Framework/Model* maturity level. Those that were prototypes, pilots, or implementations were all standalone PHR systems.

In the majority (n=45) of the articles, the patient was the data owner. Of the remaining articles, providers were data owners in 9 of them, whereas 2 had both patients and providers as owners. It was unclear who the data owner was in the last 2 articles.

In most articles, both patients and providers had read and write abilities. Most blockchain PHRs granted providers with both read and write abilities (n=40), and only 4 blockchain PHRs did not grant providers any read or write abilities. Table 5 is a matrix representing the distribution of read and write capabilities for patients and doctors among the various articles and article codes refer to article identifier in Table 3.

**Table 5:** Matrix of various read and write models among the blockchain personal health records reviewed.

Provider	Patient		
	Read only	Write only	Read and write
Read only	—^a^	—	A04, A15, A32, A42, A48, A50, A52, A58
Write only	A07, A14, A18, A31	—	—
Read and write	A01, A05, A08, A10, A12, A17, A19, A20, A23, A24, A27, A28, A30, A33, A40, A41, A43, A47, A49, A56	A25, A51	A02, A03, A06, A09, A11, A21, A22, A26, A34, A35, A37, A38, A39, A44, A45, A46, A53, A54, A55, A57
Neither	—	A16	A13, A29, A36

^a^Not available.

Most articles did not mention the adoption of any semantic standard. For those that did, the 2 standards mentioned were Fast Healthcare Interoperability Resource (FHIR) and health level 7 (HL-7) in 5 and 2 articles, respectively. Similarly, most did not mention adopting any privacy standards. For those that did, 4 mentioned compliance with the Health Insurance Portability and Accountability Act (HIPAA), 1 with the General Data Protection Regulation (GDPR), and 1 with both the HIPAA and GDPR.

Among the blockchain PHRs that were either prototypes or implementations, 9 developed a web UI, whereas 2 had both a mobile phone application UI and a desktop UI.

### Current Limitations of and Future Directions for Blockchain PHRs

#### Current Limitations

Most of the current limitations can be grouped into 1 of the following 3 main categories: (1) scalability, (2) privacy, and (3) usability. Scalability issues pertained to the inability of blockchain PHR to store large file sizes such as medical images [44,53,54] or to the slowness in confirming transactions, especially with the incorporation of streaming data from internet of things devices [45,76].

The inability of blockchain PHRs to ensure full privacy has been highlighted in a few articles. Although records on the blockchain are encrypted, there are possible means to infer the information, such as through blockchain analysis [17,43,54]. Another privacy issue raised was the inability to erase one’s records, as blockchains are inherently immutable [17,70]. This limitation would make it difficult for blockchain PHRs to comply with privacy regulations such as the GDPR, which stipulates data subjects’ right to erasure (Article 17 of the GDPR).

One of the usability limitations was the affordability of the blockchain PHR, as each transaction typically required users to pay a transaction fee [45,71]. Another practical usability issue described by Charanya et al [84] was that, unlike conventional PHRs that had password recovery mechanisms, patients would not be able to access their records if they lost their private keys on blockchain PHRs. Incapacitated or unconscious patients also present a similar problem with blockchain PHRs that do not have built-in access control when emergency health care providers would need permission to access records.

Apart from these 3 main categories, there were other limitations inherent to certain types of popular blockchains such as Ethereum. For example, Gebremedhin [45] highlighted that Solidity (Ethereum’s programming language) was unable to implement nested string data types, whereas Kung et al [86] mentioned the need to batch upload data in a certain file format as a limitation of their Ethereum-based PHR.

#### Future Directions

The current limitations provide direction to some future work areas for blockchain PHRs. Scalability solutions have already been studied and experimented on, such as Proof-of-Authority and the novel Byzanthine fault tolerance (BFT) consensus mechanisms [44,56,67]. Other methods include enhancing the blockchain architecture through tiered-chain [40,64] or side-chain structures [57]. Although privacy solutions were more limited in our review, we came across one by Reen et al [70] who proposed storing InterPlantary Naming System records instead of the conventional hash of the medical records directly on the blockchain. In this way, users may retain the ability to revoke access to the record if desired.

Many suggestions have been made to improve the usability of the system. These suggestions could be grouped into (1) user experience, (2) integration with existing systems, and (3) compliance with regulations and development of governance processes. Table 6 summarizes the suggestions proposed in the articles reviewed.

**Table 6:** Suggestions for improving blockchain personal health record usability from the selected articles.

Suggestion			Article identifiers
**User experience**			
	Improving user interface of blockchain PHRs^a^	A10, A31, A55	
	Biometric user authentication	A40	
	Allowing next-of-kin or caregiver to access records if patient grants access or is incapable of self-access	A40	
	Incorporating incentives for users	A56	
	Incorporating analytics capabilities for personal health insights and management	A32, A53	
	Adding on payment functions for health care services	A37, A55	
**Integration with existing systems**			
	Integrating with existing EMR^b^ systems	A07, A08, A19, A20, A52,	
	Adopting health care data standards	A03, A09, A17, A53	
	Integrating with IoT^c^ devices	A32	
	Integrating with open, public blockchain systems	A17, A18, A33	
**Compliance with regulations and development of governance processes**			
	Complying with regulations on health care data privacy	A07, A18	
	Developing governance processes for the blockchain PHRs	A38	

^a^PHR: personal health record.

^b^EMR: electronic medical record.

^c^IoT: internet of things.

Apart from improving usability, another aspect of future work is the validation of blockchain PHRs. Among the areas for validation, several articles suggested data validation when data were transferred to off-chain storage [77], security validation [48,58,79], and real-world validation in terms of cost-effectiveness [52,53,71]. Validating these components would be relevant to obtain stakeholder and user confidence in deciding where to implement and adopt blockchain PHRs.

## Discussion

### Principal Findings

In this first ever systematic review on blockchain PHRs, we adopted a broad search strategy across medical and CS and engineering databases and included gray literature. We focused on the scope of blockchain PHRs to allow for more targeted data abstraction. Through our study, we found that there was a growing interest in blockchain PHRs and that the space has been steadily maturing over the past few years, albeit still much in the conceptual stage. As the space is still fairly new, a lion’s share of the research and innovation has been happening at the technical level to discover new ways to solve problems. This is evidenced by the overwhelming proportion of articles that have come from the CS and engineering domain.

One of the major areas regarding blockchain PHRs that is still undergoing much research is scalability. We came across a few ideas such as Proof-of-Authority, novel BFT consensus mechanisms, and other modified blockchain architectures such as tiered-chains and side-chains [40,44,56,57,64,67]. Apart from blockchain PHR teams working on this, the space may also benefit from parallel innovations from the larger blockchain ecosystem. As Ethereum is looking forward to a new version release (version 2), it is considering various scaling solutions, of which *rollups* is a strong contender [93]. *Rollups* solution essentially involves keeping transaction data on-chain while pushing the computational load off-chain. If adopted into Ethereum 2.0, this could automatically benefit many Ethereum-based PHRs.

Although some areas are actively evolving, others are beginning to consolidate. As found in other systematic reviews, most blockchain PHR project teams have gravitated toward Ethereum and HF as their blockchains of choice [22]. In addition, in terms of data storage, we see more projects opting for IPFS as a complementary off-chain data store for their blockchain PHRs [68,72,83,92]. Outside of this review, we are also aware that there are efforts happening in other public blockchains. An example is NEO, whose core developers are developing a similarly distributed, decentralized object storage network known as NEO file storage system (NeoFS), which will seamlessly integrate with its native blockchain [94,95]. We did not come across any NEO-based PHRs in this review. NeoFS could potentially be a game changer, so it would be interesting to track its development in this area.

In this review, we also identified some current limitations that blockchain PHRs need to address. We broadly classified them into scalability, privacy, and usability limitations. In addition to identifying the current limitations, this review also revealed some possible solutions. For example, to address the privacy issue of inferring information from chain analysis, Ray Chawdhuri [54] introduced zero-knowledge provable mixing, whereas Park et al [17] proposed the zero-knowledge succinct non-interactive argument of knowledge technique. Another example is the solution of using biometric authentication mentioned by Tian [75] to address the issues of verifiable user authentication and patients losing their private keys. Medicalchain has also described an emergency bracelet that can be scanned, giving access to essential health information in unconscious patients who are unable to access their private keys [47].

The first blockchain PHR has already been piloted, and this will undoubtedly augur a move of the space toward deployment [19]. With this in mind, blockchain PHRs will need to comply with the privacy standards within the jurisdictions they intend to become operational. In addition, to enable integration with existing health care EMR systems, it is necessary to design blockchain PHRs that follow established semantic standards such as HL-7 and FHIR. Looking further ahead, to realize true decentralization, it may be necessary to consider building a PHR atop public blockchains.

Finally, in terms of geographic interest, we found that although interest in blockchain PHR was multinational, there were obvious leaders in this space. Looking deeper among the leading countries, we noticed that since 2018 there has been an increase in publications from India, whereas those from China, South Korea, and the United States started to level off or decrease. A possible reason for this could be that in 2017, in the midst of an initial coin offering (ICO) fever that drove unusually high interest in blockchain, the latter 3 countries’ relevant authorities had issued bans or indicated legal restrictions on ICO activities with stiff penalties [96-98]. This may suggest further research into the different factors, including sociopolitical, economic, and cultural factors, which could significantly impact the development of this space. In terms of interest groups, our findings should also provide a sense of where most of the developments are occurring, and this may guide government and private sector funders in their allocation of resources.

### Limitations

We acknowledge that this review is not exhaustive and that there are many other areas that were excluded. These areas include other smart contract uses, performance evaluation, and the type of vocabulary standard such as Systematized Nomenclature of Medicine Clinical Terms, 10th revision of the International Classification of Diseases, and Logical Observation Identifiers Names and Codes. We also recognize that greater detail about the read and write models could be studied, such as their validity periods and whether other stakeholders (eg, researchers and insurance companies) were given access. Future reviews should consider delving deeper into these areas.

Furthermore, despite our best efforts to capture as much material available as possible, we are aware that the exclusion of articles whose full text was not in English would have limited the scope of this review. In addition, there may also be other developments in this space that have not been made publicly available for commercial or other reasons.

### Conclusions

This cross-disciplinary systematic review on the blockchain PHR space has revealed that as of now, much of the development is still in the conceptual stage. However, there is a trend of growth and maturation. We believe that this provides consolidated evidence for researchers to continue following this space and, more optimistically, to spur them to contribute ideas and efforts to accelerate its development. Those in the medical informatics community will undoubtedly play an increasingly larger role in the development and implementation of blockchain PHRs, especially when the need to integrate with EMR systems and adopt health care data standards becomes more prominent. In addition, as the first systematic review covering blockchain PHRs, we expect this to be an important basis for subsequent reviews to track how the space has progressed in the future.

## Appendix

Multimedia Appendix 1 List of articles with data abstracted.

Multimedia Appendix 2 Overview of the articles with the article identifier, publication year, publisher, type of article, country and interest group.

## Abbreviations

BFT: Byzanthine fault tolerance
CS: computer science
EHR: electronic health record
EMR: electronic medical record
FHIR: Fast Healthcare Interoperability Resource
GDPR: General Data Protection Regulation
HF: Hyperledger Fabric
HIPAA: Health Insurance Portability and Accountability Act
HL-7: health level 7
ICO: initial coin offering
IPFS: Interplanetary File System
NeoFS: NEO file storage system
PHR: personal health record
PRISMA: Preferred Reporting Items for Systematic Reviews and Meta-analyses
PoW: proof of work
UI: user interface
