# Supplementary material for: Blockchain Personal Health Records: Systematic Review
Source: J Med Internet Res. 2021 Apr 13;23(4):e25094. doi: 10.2196/25094 (PMC8080150; doi:10.2196/25094)
Supplement: Multimedia Appendix 2 [file jmir_v23i4e25094_app2.docx]

**Multimedia Appendix 2**

Table 1 provides an overview of the articles with the identifier, publication year, publisher, type of article, country and interest group – indexed and sorted by publication year (primary) and author name (secondary) in ascending order.

Table 1. Overview of articles included in final review.

| **Article identifier** | **Authors, Year** | **Publisher** | **Type of article** | **Country** | **Interest group** |
| --- | --- | --- | --- | --- | --- |
| A01 | Burniske et al, 2016 | None | Whitepaper | USA | General |
| A02 | McFarlane et al, 2017 | None | Whitepaper | USA | General |
| A03 | Roehrs et al, 2017 | Elsevier | Journal | Brazil | Medical |
| A04 | Badr et al, 2018 | Elsevier | Conference | Egypt | CS/Engineering |
| A05 | Boiani, 2018 | None | Thesis paper | Sweden | CS/Engineering |
| A06 | Chen et al, 2018 | Springer | Journal | China | Medical |
| A07 | Dagher et al, 2018 | Elsevier | Journal | USA | General |
| A08 | Dubovitskaya et al, 2018 | AMIA | Symposium | Switzerland | Medical |
| A09 | Gebremedhin and Angosom, 2018 | None | Thesis paper | Norway | CS/Engineering |
| A10 | Lippman et al, 2018 | IEEE | Journal | USA | CS/Engineering |
| A11 | Medicalchain team, 2018 | None | Whitepaper | Switzerland | General |
| A12 | Rouhani et al, 2018 | IEEE | Conference | Canada | CS/Engineering |
| A13 | Thwin and Vasupongayya, 2018 | IEEE | Conference | Thailand | CS/Engineering |
| A14 | Vora et al, 2018 | IEEE | Conference | India | CS/Engineering |
| A15 | Zhang and Poslad, 2018 | IEEE | Conference | UK | CS/Engineering |
| A16 | Abouzahra, 2019 | AIS | Conference | USA | CS/Engineering |
| A17 | Alkhushayni et al, 2019 | EasyChair | Conference | USA | CS/Engineering |
| A18 | Chawdhuri, 2019 | Springer | Conference | India | CS/Engineering |
| A19 | Ciampi et al, 2019 | IARIA | Conference | Italy | CS/Engineering |
| A20 | Daraghmi et al, 2019 | IEEE | Journal | Palestine | CS/Engineering |
| A21 | Donawa et al, 2019 | IEEE | Conference | USA | CS/Engineering |
| A22 | Hang et al, 2019 | MDPI | Journal | South Korea | CS/Engineering |
| A23 | Harika et al, 2019 | IOPScience | Conference | Indonesia | CS/Engineering |
| A24 | Huang et al, 2019 | IEEE | Conference | New Zealand | CS/Engineering |
| A25 | Hylock and Zeng, 2019 | JMIR | Journal | USA | Medical |
| A26 | Jiang et al, 2019 | IEEE | Conference | China | CS/Engineering |
| A27 | Koushik et al, 2019 | IEEE | Conference | India | CS/Engineering |
| A28 | Lee, 2019 | WARSE | Journal | South Korea | CS/Engineering |
| A29 | MediBloc team, 2019 | None | Whitepaper | South Korea | General |
| A30 | MediLOT team, 2019 | None | Whitepaper | Singapore | General |
| A31 | Nchinda et al, 2019 | IEEE | Conference | USA | CS/Engineering |
| A32 | Nguyen et al, 2019 | IEEE | Journal | Australia | CS/Engineering |
| A33 | Park et al, 2019 | JMIR | Journal | South Korea | Medical |
| A34 | Rajput et al, 2019 | IEEE | Journal | China | CS/Engineering |
| A35 | Reen et al, 2019 | IEEE | Conference | India | CS/Engineering |
| A36 | Sangeetha et al, 2019 | Maple Tree | Journal | India | CS/Engineering |
| A37 | Shahnaz et al, 2019 | IEEE | Journal | Pakistan | CS/Engineering |
| A38 | Shekhawat et al, 2019 | None | Report | India | CS/Engineering |
| A39 | Thwin and Vasupongayya, 2019 | Wiley | Journal | Thailand | CS/Engineering |
| A40 | Tian, 2019 | None | Thesis paper | Malaysia | CS/Engineering |
| A41 | Toshniwal et al, 2019 | IEEE | Conference | India | CS/Engineering |
| A42 | Wang S. et al, 2019 | IEEE | Journal | China | CS/Engineering |
| A43 | Wang Y. et al, 2019 | IEEE | Journal | China | CS/Engineering |
| A44 | Wu and Du, 2019 | ACM | Journal | China | CS/Engineering |
| A45 | Al Goni et al, 2020 | None | Thesis paper | Canada | CS/Engineering |
| A46 | Arunkumar and Kousalya, 2020 | Springer | Conference | India | CS/Engineering |
| A47 | Aswin et al, 2020 | Springer | Conference | India | CS/Engineering |
| A48 | Cao et al, 2020 | SAGE | Journal | China | CS/Engineering |
| A49 | Charanya et al, 2020 | IGI Global | Journal | India | CS/Engineering |
| A50 | Kavathekar and Patil, 2020 | Springer | Conference | India | CS/Engineering |
| A51 | Kim et al, 2020 | MDPI | Journal | South Korea | CS/Engineering |
| A52 | Kung et al, 2020 | Springer | Conference | Taiwan | CS/Engineering |
| A53 | Lee et al, 2020 | JMIR | Journal | Taiwan | Medical |
| A54 | Sharma S. et al, 2020 | Elsevier | Conference | India | CS/Engineering |
| A55 | Sharma Y and Balamurugan, 2020 | Elsevier | Conference | India | CS/Engineering |
| A56 | Tith et al, 2020 | Korean Society of Medical Informatics | Journal | Japan | Medical |
| A57 | Verdonck and Poels, 2020 | EasyChair | Conference | Belgium | CS/Engineering |
| A58 | Wu et al, 2020 | Springer | Conference | China | CS/Engineering |
